# Effective Innovative Models of Health Care Delivery in the Era of the COVID-19 Pandemic to Reduce Disparities in Cancer Care and for Cancer Control in Low-Middle Income Countries—South African Experience of the Cancer ECHO Model

**DOI:** 10.1200/GO.22.17000

**Published:** 2022-05-05

**Authors:** Daniel Osei-Fofie

**Affiliations:** ^1^ Kimberley Hospital, Northern Cape, South Africa

## Abstract

The Northern Cape province is the largest province in South Africa. However, it has the least resources for cancer care. Patients have to travel very long distances to access cancer care. Cancers are diagnosed in late stages due to lack of resources including trained health care personnel. There was the need for an innovative model for training health care personnel, mentorship and empowerment to enable provision of cancer care in the rural areas of the province.

**METHODS:**

In 2017, the Northern Cape Health Department had a collaboration with the Project ECHO Institute, University of New Mexico. An Immersion training in ECHO was provided in Albuquerque under the sponsorship of Bristol Myers Squibb Foundation and the Project ECHO Institute. An ECHO Hub was established at the tertiary cancer center with spoke sites in two district hospitals. The first cancer ECHO in Africa was launched at Kimberley Hospital in July 2018 to provide training and mentorship in Lung cancer and Mesothelioma care. In 2020, during the COVID-19 pandemic, the ECHO clinics were used on a more regular basis for patients' management.

**RESULTS:**

Doctors, nurses and community healthcare workers have been trained using the ECHO model to provide cancer care in all the district hospitals. The ECHO clinics have now been expanded to cover other cancers. Palliative care ECHO has also been launched to improve provision of palliative care services. There has been retention of staff in the rural areas to provide cancer care due to mentorship and empowerment using the ECHO model.

**CONCLUSION:**

ECHO is an effective innovative model to democratize knowledge and help reduce disparities and inequities in cancer care and for cancer control in low-middle income countries. In the era of the COVID-19 pandemic, ECHO clinics can assist with effective patient care.

